# Synthesis of two Hofmann-type-like clathrates, characterizations, and investigation of their structures, spectroscopic and theoretical properties

**DOI:** 10.55730/1300-0527.3802

**Published:** 2026-04-09

**Authors:** Zarife Sibel ŞAHİN, Zeki KARTAL

**Affiliations:** 1 Department of Energy Systems Engineering, Faculty of Engineering and Architecture, Sinop University, Sinop, Turkiye; 2 Retired Professor of Atomic and Molecular Physics, Kütahya Dumlupınar University, Kütahya, Turkiye

**Keywords:** Hofmann-type-like clathrates, 4-aminopyridine (4AP), IR spectra, single crystal X-Ray diffraction (SC-XRD) analysis, density functional theory (DFT), Hirshfeld surfaces analysis

## Abstract

This study concerns two Hofmann-type-like clathrates using 4-aminopyridine (4AP) as ligands and the transition metal atoms copper (Cu) and zinc (Zn). These clathrates have the structural features of heteronuclear tetracyanonickelate(II) and contain H_2_O and 4AP molecules as guest molecules, respectively. These two clathrates were characterized by infrared spectroscopy (IR), elemental analysis, and single crystal X-ray diffraction (SC-XRD) techniques. Based on elemental and spectral data, the structural formulae describing these clathrates were found to be [Cu(II)(4AP)_4_Ni(μ-CN)_2_(CN)_2_]·H_2_O (**1**) and [Zn(II)(4AP)_4_Ni(μ-CN)_2_(CN)_2_]·(4AP) (**2**). In addition, the values of some optical, electronic, and thermochemical properties of the two obtained clathrates calculated with the Gaussian 03 program were compared with each other. Some theoretically calculated NLO properties of these clathrates were compared with similar properties of urea compound calculated with the same base set. The results showed that these clathrates are compounds worth investigating in terms of their NLO properties. Furthermore, the theoretical MEP maps, UV-visible region, NMR excitation transitions and Hirshfeld surface analyses of the clathrates were examined too. And it was seen that interactions such as C···H, H···H, N···H, N-H···N and C-H···π clearly play a role in the formation of their crystal structures.

## 1. Introduction

In a reaction environment, metal atoms can interact with Lewis bases, resulting in the formation of many compounds. These metal complexes are generally called “coordination compounds.” A coordination compounds contain a central metal atom surrounded by ligand molecules with various properties [1–3]. In a coordination compound, ligands donate their lone pairs of electrons, and metal atoms provide vacant orbitals to accommodate these electrons. This situation creates coordination bonds between ligand molecules and metal ions. In coordination compounds, the total count of donor atoms directly bonded to the metal atom is called the ‘coordination number’ of the compound. Coordination complexes typically exhibit a variety of bonding capacities, commonly spanning from monocoordinated to octacoordinated geometries. While various configurations exist, coordination numbers of 2, 4, and 6 are the most frequently encountered in chemical synthesis. If a metal atom has an empty orbital, it can share the unpaired electrons in the ligand molecule. The orientation of the empty orbitals in 3D space determines the shape and type of coordination compound that will be formed (linear, square planar, tetrahedral, or octahedral etc.) [3,4]. The structural architectures of coordination complexes exhibit a broad spectrum, ranging from rudimentary linear forms to highly intricate molecular networks. Factors such as pH, temperature, density, purity, etc., of the reaction medium determine the form of a coordination compound. Therefore, new compounds with different properties can be obtained with each repetition of a chemical reaction under almost the same conditions. This is because any one or more of the environmental conditions can vary, even slightly, in each reaction. Coordination compounds, which come in many different structures, are very important as natural and industrial resources. Coordination compounds can exist as neutral, positive, or negatively charged, depending on the number of electrons they have. If a coordination compound has a positive or negative electrical charge, it is also called a “complex ion.” It is common for a coordination compound to contain varying numbers of complex ions in its structure [1–5]. If a particular moiety in a coordination compound repeats periodically in an 1D, 2D, or 3D configuration, such a structure is called a “coordination polymer” [6,7].

German chemist Karl Andreas Hofmann (1870–1940) first synthesized an interesting coordination compound named after him in 1897 [8]. This coordination compound consisted of a two-component structure: a “host structure” and a “guest molecule” of appropriate size. This new compound was referred to as a “clathrate” in the scientific literature [9,10]. The general formula of Hofmann-type clathrates is given as M(II)LM′(II)(CN)_4_.nG. Here, the letters M and M′ denote transition metal atoms with a valence of +2, the letter L denotes one bidentate or two monodentate ligand molecules, the letter G denotes the guest molecule, and “n” denotes the number of guest molecules in the clathrate [8–10]. In Hofmann-type clathrates, the ratio between M, L, and M’ for a monodentate ligand molecule is 1:2:1. If this ratio is 1:3:1 or 1:4:1, or even different, the resulting clathrate is called a Hofmann-type-like clathrate.

In the molecular architecture of 4-aminopyridine (4AP), the amino functionality is localized on the carbon atom situated diametrically across from the pyridine nitrogen. If the amino group is bonded to other carbon atoms, it is called 2-aminopyridine (2AP) or 3-aminopyridine (3AP). Aminopyridines are compounds that play a significant role in the medical and chemical fields [11–17].

Copper (Cu) is a metal with very high thermal and electrical conductivity. It is also a necessary and very important metal for vital activities in all living things [18]. Because copper is malleable, ductile, and pliable, it can easily be made into very thin wires, sheets, and ropes. Since it is softer than zinc (Zn), a copper sheet can be polished to a mirror-like finish. Although the copper belongs to the same group as gold (Au) and silver (Ag), it has lower chemical reactivity compared to them [18].

From a human health perspective, zinc metal (Zn) is not as toxic as some other metals in the same group. Zinc metal is an essential trace element for humans and all other living organisms. Adequate amounts of zinc metal are essential for cell growth and division, i.e., the maintenance of life, in all organisms [18].

Nickel (Ni) is a transition metal with strong magnetic properties that is very resistant to external influences. It is added to other elements such as iron and copper to make them more durable. Nickel is an excellent conductor of electricity and heat. The nickel atom can have valences ranging from −1 to +4 in the compounds it forms. However, the most common valence in its compounds is +2. The geometric arrangement of the nickel atom in its compounds can be octahedral, trigonal bipyramidal, tetrahedral, and square planar, depending on its valence. Furthermore, nickel is an essential nutrient for all plants [18].

Cyanonickelate compounds are generally anions composed of nickel atoms and cyanide ligands. One of the most important cyanonickelate compounds is tetracyanonickelate, which contains four cyanide groups per nickel atom [Ni(CN)_4_]^2-^. The most characteristic and obvious vibration peak of cyanonickelate ions is the C≡N stretching peak. Tetracyanonickelate has a square planar geometry and is diamagnetic [19].

In our previous study, we obtained new Hofmann-type and Hofmann-type-like clathrates in crystalline form using 4AP as the ligand molecule and copper metal atom. The formula of the Hofmann-type-like clathrate obtained in this study was [Cu(II)(4AP)_4_Ni(μ-CN)_2_(CN)_2_]·H_2_O. This compound had a different structure than the clathrate targeted to be obtained. Some experimental and theoretical results related to it have been published [15].

Our aim in this study was to obtain new clathrates similar to the Hofmann-type-like clathrate given by the formula [Cu(II)(4AP)_4_Ni(μ-CN)_2_(CN)_2_]·H_2_O. As a result, we obtained a Hofmann-type-like clathrate containing 4AP molecule as both ligands and guests and a zinc (Zn) metal atom, a [Ni(CN)_4_]^2-^ ion.

In this study, the chemicals ZnCl_2_, 4AP, and K_2_Ni(CN)_4_.H_2_O were used in a 1:6:1 ratio to obtain the crystalline form of a Hofmann-type-like clathrate. Here, the 4AP molecules were used as both the maximum number of ligands and the guest molecules. Consequently, a crystalline clathrate with the explicit chemical formula [Zn(II)(4AP)_4_Ni(μ-CN)_2_(CN)_2_]·(4AP) was obtained.

In this study, new theoretical properties of the Hofmann-type-like clathrate with the formula [Cu(II)(4AP)_4_Ni(μ-CN)_2_(CN)_2_]·H_2_O [15], for which some theoretical and experimental data have been published previously, will be given. Furthermore, various theoretical and experimental data of the newly obtained Hofmann-type-like clathrate with the formula [Zn(II)(4AP)_4_Ni(μ-CN)_2_(CN)_2_]·(4AP) will be compared with its data. Hereinafter, clathrate [Cu(II)(4AP)_4_Ni(μ-CN)_2_(CN)_2_]·H_2_O (1) and clathrate [Zn(II)(4AP)_4_Ni(μ-CN)_2_(CN)_2_]·(4AP) (2) will be referred to with their abbreviations.

Electrical and thermal properties of related Hofmann-type-like clathrates having two different guest molecules were also calculated theoretically with Gaussian 03 software package. In addition, all interactions, charge distributions and crystal voids taking part in the formation of these clathrates were calculated from their cif files with the help of CrystalExplorer program.

## 2. Experimental

### 2.1. Materials

For the preparation of the desired compounds, the following analytical-grade reagents were procured: 4AP (C_5_H_6_N_2_), (Sigma Aldrich, 99%); K_2_[Ni(CN)_4_]·H_2_O, (Fluka, Buchs, Switzerland; 96%); anhydrous copper(II) chloride (CuCl_2_) (Fluka, 99%); anhydrous zinc(II) chloride (ZnCl_2_) (Fluka, 97%) and aqueous ammonia solution (NH_3_, Merck, 25%).

### 2.2. Syntheses of compounds

The synthesis of compound 1 is given in the previously published article [15]. Compound 2 was synthesized according to the following experimental procedure:

Initial dissolution of 1 mmol K_2_[Ni(CN)_4_]·H_2_O (0.259 g) in 10 mL distilled water was followed by the gradual addition of 6 mmol 4AP (0.564 g) under continuous stirring. In this study, the 4AP molecule was used as both a ligand and a guest molecule in the compound, so it was taken in six times more amount than the other components. After solubilizing 1 mmol anhydrous ZnCl_2_ (0.137 g) in 5 mL distilled water, the aliquot was blended with the initial mixture and maintained under constant stirring at 60 °C for 20 minutes.

The blend was treated with dropwise ammonia until transparency was reached, and subsequently maintained at a steady 60 °C with agitation for 20 minutes. Post-filtration, the solution underwent a slow evaporation process in a controlled ambient environment. Purple-hued, transparent crystalline solids were harvested following a maturation period of roughly three weeks.

### 2.3. Instrumentation

The vibrational properties of the synthesized Hofmann-type clathrates were investigated via FT-IR spectroscopy using a Bruker Optics Vertex 70 spectrometer. While elemental quantification of C, H, and N was performed with a LECO CHNS-932 analyzer, the metallic content (Ni, Cu, and Zn) was determined through ICP-OES (Perkin-Elmer Optima 4300 DV). For structural elucidation, X-ray diffraction data were collected on a Bruker D8-QUEST diffractometer equipped with a graphite-monochromated Mo-*K*_α_ radiation source (λ = 0.71073 Å).

Table S1 presents a comparison between the experimental and theoretical elemental compositions for the crystalline Hofmann-type-like clathrates. These values encompass all elements identified within the synthesized structures. Theoretical and experimental values of the elements in the structures of each newly obtained Hofmann-type-like clathrate are quite compatible with each other. From Table S1 and the examination of the SC-XRD structure analyses of the compounds, it is seen that compounds 1 and 2 are Hofmann-type-like clathrates.

The host structures of these two Hofmann-type-like clathrates are very similar. The only differences between them are the presence of transition metals (Cu and Zn) and guest molecules (H_2_O and 4AP).

## 3. Results and discussion

### 3.1. Crystallographic analyses of compounds 1 and 2

The positional parameters of C-attached H atoms were constrained using the riding atom approximation (C-H = 0.93 Å); in contrast, all other hydrogen atoms in the structure were refined isotropically without restraints. We used these procedures for our analysis: solved by direct methods; SHELXS-2013 [20]; refined by full-matrix least-squares methods; SHELXL-2013 [21]; data collection: Bruker APEX2 [22]; molecular graphics: MERCURY [23]; solution: WinGX [24]. Detailed experimental conditions for data collection and structure solution procedures are provided in Table S2.

#### 3.1.1. Compound 1

The majority of the crystal structure data for this compound belongs to compound 2, CCDC number 1565193, in reference [15]. Newly obtained data for this compound are listed below.

The Cu(II) and Ni(II) transition metal ions are bridged by cyanide ligands to generate a 1D coordination polymer running parallel to the [100] direction (Figure S1), with the distances between the Cu···Ni metal ions in this polymeric structure being 5.388 and 5.403 Å. Within the crystal lattice, N6 participates in hydrogen bonding with N1 at (x-1/2, y-1/2, z), facilitating the propagation of a C(12) chain in the [110] direction [See Figure 3 in Reference 15]. In a perpendicular fashion, N8 donates to the N3 atom at (-x+3/2, y+1/2, −z+1/2), forming a longitudinal C(12) chain along [010]. This pattern is mirrored by N10 and N12, which generate analogous C(12) chains through their respective symmetry-related nitrogen acceptors. Ultimately, the coalescence of these hydrogen-bonded networks produces distinct ring motifs as illustrated in Figure S1.

#### 3.1.2. Compound 2

**Figure 1:**
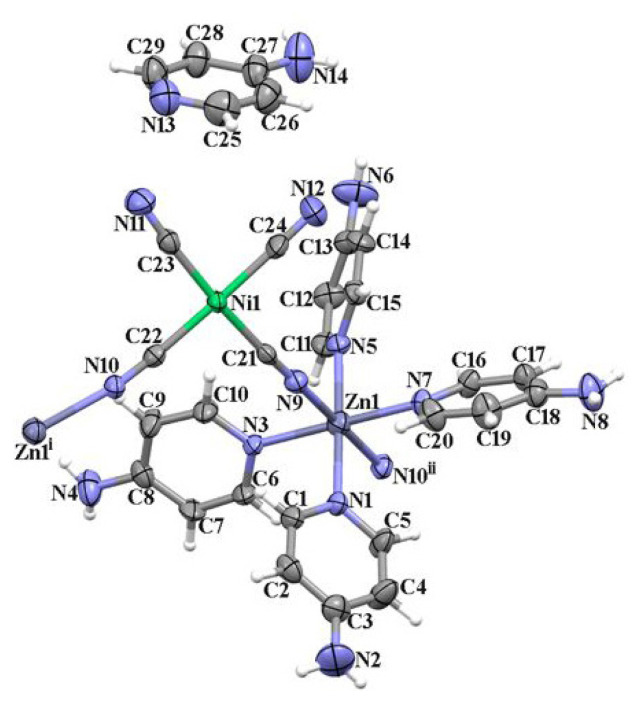
The molecular structure of the compound 2 showing the atom numbering scheme. Displacement ellipsoids of non-H atoms are drawn at the 50% probability level. [Symmetry codes: (i) *x*+1/2, −*y*+1/2, *z*+1/2; (ii) *x*–1/2, −*y*+1/2, *z*–1/2]

The SC-XRD study shows that the heterometallic compound 2 has 1D coordination polymer. The asymmetric unit of the heterometallic compound 2 consists of one Zn(II) ion, one Ni(II) ion, four cyanide ligands, four coordinated 4AP ligands and one non-coordinated 4AP ligand (Figure 1). The compound 2 has crystal structure in the 
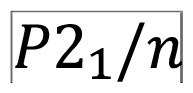
 space group. The Zn(II) ion is coordinated by four nitrogen atoms [Zn-N distances ranged between 2.093(4) - 2.145(4) Å] from 4AP ligands and two nitrogen atoms [2.113(4) Å and 2.122(4) Å] from cyanide ligands (Table 1), thus showing an octahedral coordination geometry. A similar situation was observed for the Cu(II) ion in compound 1. The Cu(II) ion there is coordinated in a distorted octahedral geometry by four N atoms of the 4AP ligands [Cu-N distances ranged between 2.026(7) and 2.041(8) Å] and two N atoms of the cyanide ligands [Cu-N distances ranged between 2.692(4) and 2.695(4) Å] [15]. The Ni(II) ion is coordinated by four carbon atoms {Ni-C distances ranged between 1.854(6) - 1.875(5) Å [15, 17]} from cyanide ligands, thus showing a square planar coordination geometry. The same phenomenon was observed for the Ni(II) ion in compound 1. The Ni(II) ion there is coordinated by four carbon atoms of the cyanide ligands [Ni-C bond range between 1.848(11) and 1.867(8) Å] in a square planar coordination geometry [15].

**Figure 2:**
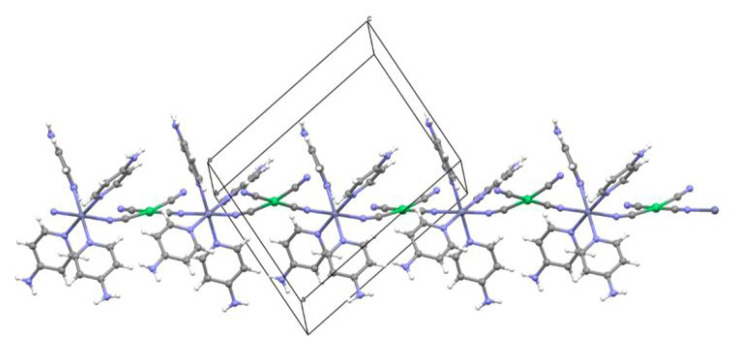
An infinite 1D supramolecular network in compound 2.

The Zn(II) and Ni(II) ions are bridged by cyanide ligands to generate 1D coordination polymer which is running parallel to the [101] direction (Figure 2), with the Zn···Ni separations are 5.059 and 5.109 Å.

The most remarkable property of the compound is the presence of N-H···Ni interaction between Ni(II) ion and H2B atom of the 4AP ligand: Ni1···H2B = 2.84 Å, Ni1···N2 = 3.0463(5) Å and N2-H2B···Ni1 = 97.53(2)°, respectively. The molecules are connected by N-H···N hydrogen bonds and C-H···π interaction (Table 2). Atom N4 atom acts as hydrogen bond donor to atom N11 in the molecule at (3/2-x, y-1/2, 3/2-z), forming a C(12) chain which is running parallel to the [010] direction (Figure S2). N6 atom acts as hydrogen-bond donor to atom N12 in the molecule at (1-x, 1-y, 1-z), forming a centrosymmetric $R_{2}^{2}(24)$ ring centered at (1/2, 1/2, 1/2) (Figure S3). The combination of other hydrogen bonds produces $R_{4}^{4}(36)$ ring (Figure S4).

A supramolecular C(7) chain is established along the [101] vector through a C-H···π contact, where the C4 atom (x, y, z) targets the N5/C11-C15 pyridine system situated at (1/2+x, 1/2-y, -1/2+z). This directional interaction, illustrated in Figure S5, facilitates the longitudinal propagation of the molecular assembly.

### 3.2. Experimental FT-IR investigations of compounds 1 and 2

**Figure 3:**
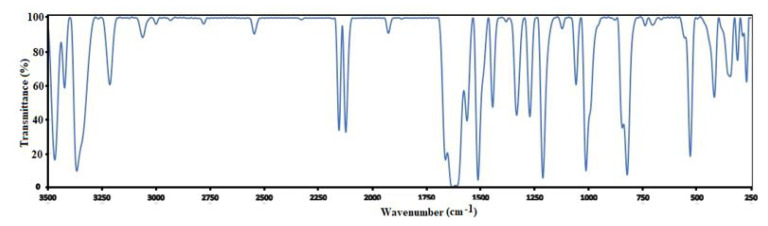
The FT-IR spectrum of compound 2.

The FT-IR spectra of the 4AP ligand molecule and compound 1 were presented in our previous article [15]. The FT-IR spectrum of compound 2 is also given in Figure 3. The highly similar FT-IR spectra of both compounds clearly suggest their structural similarity. The spectral data of this compound will be analyzed as both the ligand and guest molecule states of 4AP and the vibrations of the [Ni(CN)_4_]^2−^ anion, respectively.

In the IR spectra of the free 4AP ligand molecule and compound 1, symmetric and asymmetric stretching vibrations are each observed as a single intense peak [see the relevant section in reference 15]. However, in the IR spectrum of compound 2, four peaks are observed in this region, each close to the other. The first two of these peaks (at higher wavenumbers) belong to asymmetric stretching vibrations of the guest and ligand 4AP molecules, respectively. The other two peaks (at lower wavenumbers) belong to symmetric stretching vibrations, respectively. Some of the important peaks and their labels for compounds 1 and 2 are listed in Table 3 for easier comparison.

#### 3.2.1 Investigation of experimental vibrations of the 4AP ligand and guest molecule in compounds

According to the crystalline structure data of compound 2, 4AP molecule is present in this structure as both a guest and a ligand. The charge distribution of a molecule in its free state differs from that of its compounded state. The charge value of a functional group in its free state is greater than the charge value in its compounded state. Therefore, the vibration peaks of a functional group in the vibration spectrum of a molecule can appear at different wavenumbers depending on whether it is free or bound.

According to this explanation, the stretching vibration peaks at higher wavenumbers in the IR spectrum of compound 2 belong to the guest 4AP molecule, while the peaks at lower wavenumbers belong to the ligand 4AP molecule. In particular, it is clearly seen from both Figure 3 and Table 3 that the ν_as_(NH_2_), ν_s_(NH_2_) and ν(C-NH_2_) stretching vibrations of the 4AP molecule and the δ(NH_2_) vibration of NH_2_ are split into two. However, since the 4AP molecule is only present as a ligand in compound 1, there is no splitting in the modes in question.

In fact, vibration modes that occur around approximately the same wavenumber for the same or different molecules present as both ligands and guests in compounds are observed as a single, broader peak rather than as two separate, narrow peaks. Furthermore, examination of Table 3 indicates that many vibration modes of the 4AP molecule change relative to its free state upon compound formation. With compound formation, some vibration modes shift slightly to either higher wavenumbers or lower wavenumbers (+Δ or −Δ) due to changes in coupling constants (increases or decreases). Columns showing the shifts in the wavenumbers of some vibrational modes of the 4AP ligand molecule due to compound formation have been added to Table 3. Among the wavenumbers shifts of the 4AP ligand molecule in compounds 1 and 2, those with the largest values are marked in bold in Table 3.

In the IR spectrum of the free 4AP molecule, the ν_as_(NH_2_) vibration mode occurring at wavenumber 3430 cm^−1^ was observed at wavenumber 3479 cm^−1^ in compound 1, while it was split into two at wavenumbers 3469 and 3424 cm^−1^ in compound 2. The shifts of this vibration mode in compounds 1 and 2 are 49 with 39 and -6 cm^−1^, respectively.

In the IR spectrum of the free 4AP molecule, the ν_s_(NH_2_) vibration mode occurring at wavenumber 3303 cm^−1^ was observed at wavenumber 3342 cm^−1^ in compound 1, while it was split into two at wavenumbers 3367 and 3215 cm^−1^ in compound 2. The shifts of this vibration mode in compounds 1 and 2 are 39 with 64 and -88 cm^−1^, respectively.

In the IR spectrum of the free 4AP molecule, the δ(NH_2_) vibration mode occurring at wavenumber 1649 cm^−1^ was observed at wavenumber 1641 cm^−1^ in compound 1, while it was split into two at wavenumbers 1663 and 1634 cm^−1^ in compound 2. The shifts of this vibration mode in compounds 1 and 2 are -8 with 14 and -15 cm^−1^, respectively.

Further examination of Table 3 reveals that wavenumber shifts or splitting events also occurred in other vibration modes of the 4AP molecule. These shifts in the vibration modes of the 4AP molecule are greater in compound 2 compared to those in compound 1. This may be due to the presence of the 4AP ligand molecule as both a ligand and a guest in compound 2.

#### 3.2.2. IR active vibrations of [Ni(CN)_4_]^2−^ ion in compounds

In this study, the frequencies of the Ni(CN)_4_ group vibrations in compounds 1 and 2 were assigned based on the work of McCullough et al. [25]. The IR-active vibration modes of the Ni(CN)_4_ ion in compounds 1 and 2 are given in Table 4.

In the IR spectrum of the chemical substance K_2_[Ni(CN)_4_]·H_2_O, one of the fundamental materials used in the preparation of related compounds, the [ν(C≡N), E_u_] vibrational mode is observed at a wavenumber of 2122 cm^−1^. Generally, the peak of the ν(C≡N) vibration mode in cyanide compounds is narrow, sharp and quite intense, the most characteristic peak. This vibration mode exhibits an interesting feature by splitting into two in the IR spectra of the compounds 1 and 2. This splitting occurs because opposite two of the four C≡N groups in the compounds participate in the formation of the 1D chain structure and act as bridges, while the other opposite two C≡N groups remain as terminal ends. Of these split vibration bands, those showing bridge properties are observed at wavenumbers of 2170 and 2155 cm^−1^ and those in the terminal state are observed at wavenumbers of 2136 and 2124 cm^−1^ in compounds 1 and 2, respectively. The shift amounts of the bridge states of this vibration mode in compounds 1 and 2 are 48 and 33 cm^−1^, respectively, while the shift amounts of the terminal states are 14 and 2 cm^−1^, respectively.

In the IR spectrum of the compound K_2_[Ni(CN)_4_]·H_2_O, the ν_9_(Ni–CN), E_u_ vibration mode, which occurs at a wavenumber of 544 cm^−1^, is observed at wavenumbers of 565 and 560 cm^−1^ in compounds 1 and 2, respectively [15]. The shift amounts of this vibration mode in compounds 1 and 2 are 21 and 16 cm^−1^, respectively.

In the IR spectrum of compound K_2_[Ni(CN)_4_]·H_2_O, the π(Ni–CN), A_2u_ vibration mode, which occurs at a wavelength of 442 cm^−1^, was observed at a wavelength of 448 cm^−1^ in compound 1 [15], but this vibration mode was not observed in compound 2. This may be due to a change in the symmetry of this mode in compound 2 or a loss of IR activity. The shift amount in this vibration mode is 6 cm^−1^ in compound 1.

In the IR spectrum of the compound K_2_[Ni(CN)_4_]·H_2_O, the δ(Ni–CN), E_u_ vibration mode, which occurs at a wavenumber of 420 cm^−1^, is observed at wavenumbers of 417 and 421 cm^−1^ in compounds 1 and 2, respectively. The shift amounts of this vibration mode in compounds 1 and 2 are -3 and 1 cm^−1^, respectively.

According to the data in Table 4, the largest changes in the IR active vibration modes of the Ni(CN)_4_ ion due to compound formation occurred in compound 1, where the Cu metal atom is located.

New M-N (M = Cu and Zn) stretching vibrations resulting from the formation of compounds were obtained in their IR spectra at (480 vw, 355 m and 295 m) cm^−1^ [15] and (486 vw, 348 m and 272 m) cm^−1^ for compounds 1 and 2, respectively. Similar phenomena were also observed by other researchers [14,15 and 26].

## 4. Theoretical computational studies on compounds

**Figure 4:**
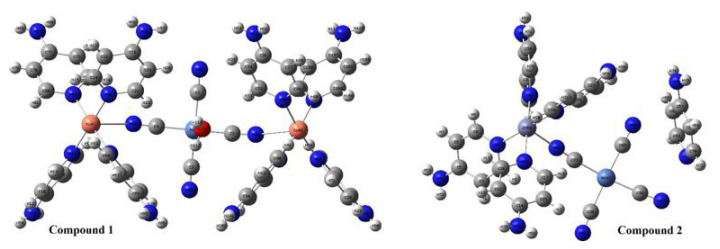
Atomic numbering used in theoretical calculations of compounds.

Since the crystalline compounds in question contain Ni, Cu, and Zn transition metal atoms, their structural parameters were calculated in the Gaussian 03 program [27], the LanL2MB basis set with the DFT/B3LYP method [28–31]. Some electronic, magnetic, and thermochemical values of the compounds were obtained from the calculation results [27]. The GaussView 4.1 visualization program was used for all these tasks [32]. The atomic numbering used in the theoretical calculations for compounds 1 and 2 is shown in Figure 4.

### 4.1. Mulliken atomic charge values of compounds

The charges defined as “Mulliken charges” in a compound are the positive or negative electric charges possessed by each atom, which maintain the compound in electrostatic equilibrium. The types, locations, and values of these Mulliken charges are the basis of the electromagnetic properties of that compound. The values of the Mulliken charges in the resulting compounds are given in Table S3 in terms of the free electron charge (e).

A careful examination of Table S3 reveals the following conclusions.

- All H atoms in both compounds are positively charged.
- The largest positively charged H atoms in both compounds are in the NH_2_ group. The smallest positively charged H atoms are the H atoms in the pyridine ring and H_2_O.
- In both compounds, the C atoms are present with both positive and negative charges.
- The largest positively charged C atoms are those bonded to the NH_2_ group. The smallest positively and negatively charged C atoms are those bonded to the N atom in the pyridine ring.
- All N atoms in both compounds are negatively charged.
- The N atoms with the highest negative charge are in the NH_2_ group, the N atoms with the median negative charge are in the C≡N group, and the N atoms with the least negative charge are in the pyridine ring.
- Cu and Zn atoms in compounds have a positive electric charge.
- The O atom in compound 1 has a negative electric charge.
- The Ni atoms in compounds 1 and 2 have the maximum negative electric charge.

### 4.2. HOMO-LUMO energy levels of compounds

All atoms have atomic orbitals at various energy levels where each electron has the highest probability of being found. When multiple atoms form a compound, these atomic orbitals combine to form molecular orbitals.

**Figure 5:**
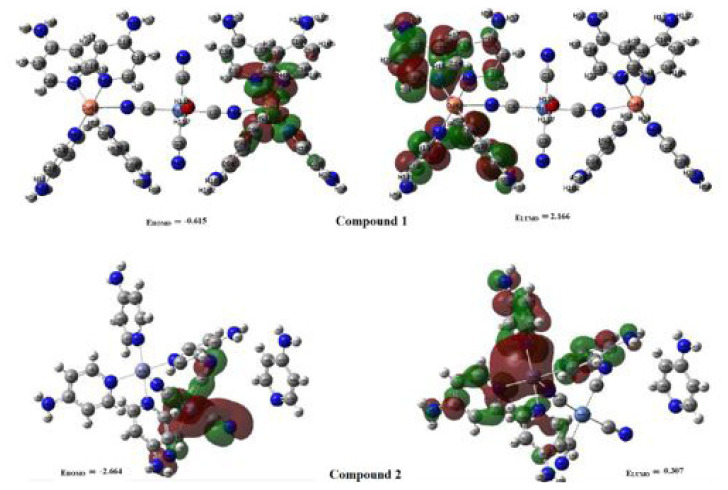
The HOMO and LUMO energy graphs of compounds in electronvolt unit (eV).

The highest-energy molecular orbital filled with electrons is called the “HOMO,” and the lowest-energy completely empty orbital is called the “LUMO.” The energy difference between the HOMO and LUMO states determines the chemical stability of the compound and the color of its solutions. The HOMO-LUMO energy graphs of the compounds were obtained using the DFT/B3LYP method with the LanL2MB basis set in the Gaussian 03 program [27–32]. The graphs of the HOMO-LUMO states of the compounds are shown in Figure 5.

According to Figure 5, it is clearly seen that the charge and energy distributions in the HOMO state of compound 1 are localized on the Cu atom and some atoms of the 4AP ligand molecule bound to it, located to the right of the Ni atom. The charge and energy distributions in the LUMO state of the same compound are localized on the Cu atom and some atoms of the 4AP ligand molecule bound to it, located to the left of the Ni atom. However, in this case, slightly more atoms of the 4AP ligand molecules are involved in this process than in the HOMO state.

In the HOMO state of compound 2, the charge and energy distributions are generally concentrated on the terminal C≡N groups of the Ni(CN)_4_ ionic structure and the Ni atom. The charge and energy distributions in the LUMO state of the same compound are located entirely on the Zn atom and the atoms of the four 4AP ligand molecules bound to it.

Theoretical molecular orbital (MO) calculations for the compounds were performed using the DFT/B3LYP method [27–32] and the LanL2MB basis set. As a result, compound 1 has a total of 772 MOs, of which 274 are occupied and 498 are vacant. Compound 2 has a total of 267 MOs, of which 166 are occupied and 101 are vacant.

Equations (1–5) are used to calculate the electrochemical properties of the compounds from their HOMO and LUMO values [16, 33–38].


(1) $$\Delta \text{E}={\text{E}}_{\text{LUMO}}-{\text{E}}_{\text{HOMO}}\;(\text{Energy gap value})$$

(2) χ=(I+A)/2=-μ   (Electronegativity,Negative chemical potential)

(3) η=(I-A)/2         (Chemical hardness)

(4) S=1/2η         (Chemical softness)

(5) $$\omega =\mu^{2}/2\eta \;\;\;(\text{Electrophilicity index})$$

These values calculated theoretically from Equations (1 – 5) are given in Table 5 in both (au) and (eV) terms.

Examination of Table 5 reveals that ΔE_2_ > ΔE_1_. Accordingly, compound 2 will exhibit higher kinetic stability and lower chemical reactivity than compound 1. A comparison of the electronegativity values of the compounds shows χ_1_ > χ_2_. Furthermore, χ_1_ is positive, while χ_2_ is negative. The chemical hardness values of the compounds are ordered as η_2_ > η_1_, and both values are negative. The chemical softness and electrophilicity index values of the compounds are as follows: S_1_ > S_2_ and ω_1_ > ω_2_, all negative values.

### 4.3. Calculated NLO properties of compounds

In a compound, the distribution of positive and negative electric charges constitutes its electric dipole moment (). The dipole moment is a 3D vector quantity. If a dipole moment changes in magnitude or orientation with time, the electric charges in the compound are moving. If a compound is in electrostatic equilibrium, the dipole moment remains constant and does not change with time. If this occurs, the spatial position of that compound is definite.

Applying an external electric field to a molecular system induces a migration of internal charges, which temporarily offsets the original electrical neutrality. This shift continues until the compound reaches a revised state of electrostatic stability. To quantify this susceptibility to charge redistribution, the term ‘polarizability’ (α) is employed. While the concept encompasses several specialized interpretations, the ‘average polarizability’ (α_0_) remains the standard metric in most technical and computational contexts.

Apart from the above-mentioned electrical properties of a compound, there are other electrical properties defined by the symbols Δα, β_0_ and γ [33–38]. All components of the electrical properties of the relevant compounds were obtained theoretically in the DFT/B3LYP method with the LanL2MB basis set [27–32]. Theoretically obtained components were used in formulas (6–13) with the ZEKA utility program [39] to calculate all electrical properties of the compounds.

The obtained results are given in Table 6 in electrostatic units (esu) using the necessary conversion factors [40,41].


(6) $$\mu =\sqrt{\mu_{x}^{2}+\mu_{y}^{2}+\mu_{z}^{2}}\;(\text{Dipole moment})$$

(7) $$\alpha_{0}=\frac{\alpha_{xx}+\alpha_{yy}+\alpha_{zz}}{3}\;(\text{Mean polarizability})$$

(8) $$\Delta \alpha =\sqrt{\frac{{\left(\alpha_{xx}-\alpha_{yy}\right)}^{2}+{\left(\alpha_{yy}-\alpha_{zz}\right)}^{2}+{\left(\alpha_{zz}-\alpha_{xx}\right)}^{2}+6(\alpha_{xy}^{2}+\alpha_{xz}^{2}+\alpha_{zy}^{2})}{2}}\;(\text{Anisotropies of polarizability})$$

(9) $$\beta_{x}=\beta_{xxx}+\beta_{xyy}+\beta_{xzz}\;\;\;(\text{x component }\beta_{0})$$

(10) $$\beta_{y}=\beta_{yyy}+\beta_{xxy}+\beta_{yzz}\;\;\;(\text{y component of }\beta_{0})$$

(11) $$\beta_{z}=\beta_{zzz}+\beta_{xxz}+\beta_{yyz}\;\;\;(\text{z component of }\beta_{0})$$

(12) $$\beta_{0}=\sqrt{\beta_{x}^{2}+\beta_{y}^{2}+\beta_{z}^{2}}\;\;\;(\text{First-order static hyperpolarizability})$$

(13) $$\gamma =\frac{1}{5}\left\{\gamma_{xxxx}+\gamma_{yyyy}+\gamma_{zzzz}+2\left[\gamma_{xxyy}+\gamma_{xxzz}+\gamma_{yyzz}\right]\right\}\;(\text{Second-order static hyperpolarizability})$$

According to Table 6, the relationships between some electrical properties of the compounds can be given as follows.

- *μ*_2_ ≅ 3.247*μ*_1_
- Δα_2_ ≅ 1.853Δα_1_
- *α*_0_2__ ≅ 0.877*α*_0_1__
- *β*_0_2__ ≅ 3.225*β*_0_1__
- *γ*_2_ ≅ 0.500*γ*_1_

According to these results, only the α_0_ and γ values of compound 1 are greater than the same values of compound 2, while the μ, Δα, and β_0_ values of compound 2 are greater than the same values of compound 1. In other words, compound 2 can be expected to be more responsive to electrical effects than compound 1.

Based on theoretical calculations using the same basis set, the β_0_values of complexes 1 and 2 are approximately 48.96 and 157.90 times higher than that of urea, respectively. Simultaneously, the γ values of complexes 1 and 2 are approximately 276.47 and 138.32 times higher than that of urea, respectively. Therefore, Hofmann-type-like complexes and clathrates can be very interesting new examples for researchers interested in the nonlinear optical properties of compounds.

Additionally, some thermochemical properties of the compounds, which play an important role in their interactions with other compounds in their environment, were calculated using the ZEKA utility [39], and the results are listed in Table S4.

As seen in Table S4, while the values of compounds’ most thermochemical properties vary directly proportional to the number of atoms in the compound, only the values of the compounds’ rotational constants vary inversely proportional to the number of atoms in the compound.

### 4.4. Molecular Electrostatic Potentials (MEPs) of Compounds

**Figure 6:**
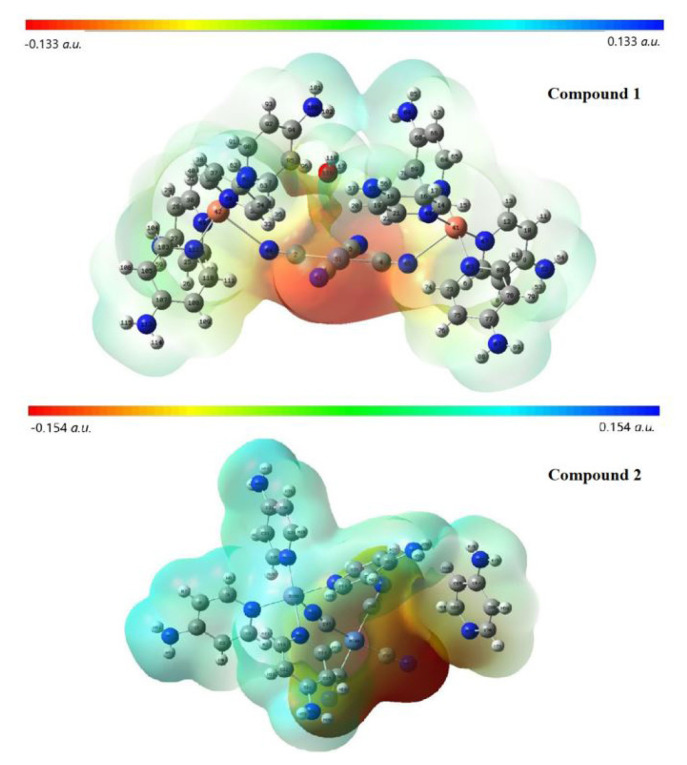
MEP maps of compounds calculated at DFT/B3LYP/LanL2MB base set.

Reactive sites in the structures of compounds are visualized by their molecular electrostatic potential (MEP) maps. The MEP maps of the relevant compounds were obtained using the B3LYP/LanL2MB method (Figure 6). In a compound’s MEP map, red and yellow areas represent areas with negative electrostatic potential energy, which are associated with electrophilic reactivity, while blue and white areas represent low electron density and partially positive regions, which are associated with nucleophilic reactivity.

According to Figure 6, the negatively charged regions generally found around Ni(CN)_4_ ions in compounds are regions of high electronegativity. Furthermore, the blue regions formed around the hydrogens bonded to the nitrogen and carbon atoms in the ligand molecules indicate potential nucleophilic sites. The guest water and 4AP molecules in compounds 1 and 2, respectively, appear to have distinctive blue and red regions independent of their surroundings.

### 4.5. Theoretical UV-visible spectra calculated from structure data of compounds

**Figure 7:**
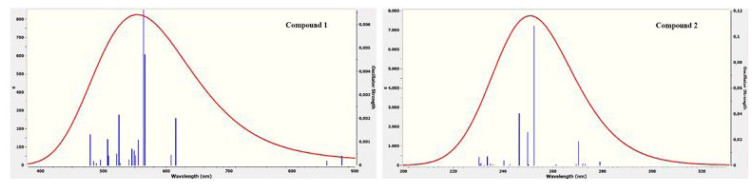
Theoretically calculated UV-visible absorption spectra of compounds in water solvent.

Theoretically, UV-visible region electronic transitions were calculated for compounds 1 and 2 (N_state_ = 20) under the conditions [scrf = (iefpcm, solvent = water)] using the TD-SCF, B3LYP/LanL2MB basis set. Graphs of these electronic transitions were obtained from the relationship between wavelengths (λ, in nm) and oscillation strengths (f, unitless) (Figure 7) [42–44].

Theoretically calculated excitation transitions for compounds can occur at different wavelengths and intensities, depending on their structural, electronic, and other properties. These excitation transitions and their associated information are given below.

A total of 86 theoretical excitation transitions were calculated for compound 1. All of these are in the group of allowed transitions. A total of 61 theoretical excitation transitions were calculated for compound 2. Four of these are in the group of forbidden excitation transitions, while 58 are in the group of allowed transitions. All UV-visible excitation transitions play a critical role in the formation of a compound. Table S5 was created by taking as an example only the largest coefficient of the allowed ones from each state of the excitation of the compounds. These theoretically calculated exemplary excitation transitions for the compounds and their various properties are listed in Table S5.

According to Table S5 and Figure 7, all excitation transitions for compound 1 occur in the visible region, while all excitation transitions for compound 2 occurred in the UV region. Similar results were obtained for each new UV-visible excitation transition calculation repeated with a different N_state_ for both compounds.

According to experimental and theoretical data, the transitions between approximately 210 and 290 nm in the UV-visible excitation transitions of a compound are due to the π → π* and n → π* transition types. The transitions between approximately 300 and 400 nm are considered to correspond to charge transitions between the atoms of the ligand molecule and the transition metal (L→M), and the transitions between approximately 400 and 800 nm are considered to correspond to the interaction of light with the compounds [42–44].

According to these explanations, all transitions in compound 1 correspond to the interaction of light with this compound, while all transitions in compound 2 correspond to the π → π* and n → π* transitions.

An examination of Table S5 reveals the following conclusions:

- The π → π* and n → π* transitions in compound 2 occurred in the wavelength ranges of 230.51 – 278.39 nm. However, no such transition was observed in compound 1.
- The L → M charge transitions were not observed in either compound.
- The visible region transition occurred in the wavelength range of 479.06 – 615.04 nm in compound 1.
- The two transitions occurring at wavelengths of 855.38 and 879.39 nm in compound 1 are thought to be related to near-IR vibrations.

**Figure 8:**
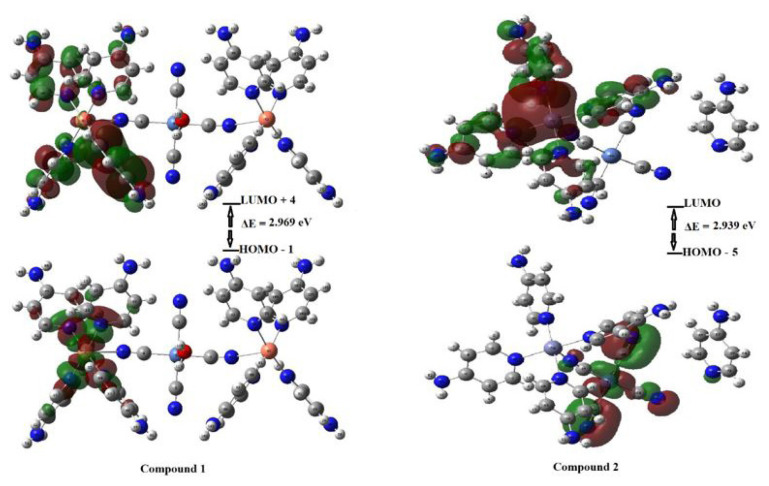
The charge distribution graphs of the UV-visible transitions with the highest oscillation strengths of compounds 1 and 2.

The charge distribution graphs for the transitions from orbital 258 to orbital 264 for compound 1 and from orbital 161 to orbital 167 for compound 2 are shown as examples in Figure 8. These transitions have the highest “f” oscillation strengths for their respective compounds.

The reason why the energy values required for these UV-visible transitions of the compounds are very close to each other is that their structures are very similar.

### 4.6. Theoretical NMR analysis of compounds

In theoretical and experimental NMR spectra of compounds, the chemical and magnetic shift values of the elements relative to their free states provide undeniable evidence of the formation of the compound. Shift values for the elements ^1^H and ^13^C are the easiest tools to indicate structural changes in the compound [45–47]. When all shift values are interpreted, crucial information about the molecular structure of that compound is obtained. Using data from the compounds’ cif files, their NMR spectra were calculated using the GIAO approach [45–48] with the Gaussian 03 program [27–32].

The signal associated with an isotropic chemical shift of an element in a molecular structure is determined by the structural electronic environment of that element. This value is independent of the element’s position within the molecular structure. When an external magnetic field interacts with a molecular structure, the charge of a particular atom in resonance is called an anisotropic displacement. The magnitude of this anisotropic displacement depends on the atom’s spatial environment within the molecular structure.

Isotropic chemical shift values between -0.05 and +0.05 ppm for elements of the same type were considered as a single group, and a “mean isotropic shift value” was assigned to that group. The number of atoms in a group with the same mean isotropic shift value indicates the “degree of degeneracy” of that group.

The anisotropic shift value of any element in a compound depends not only on its location in the compound but also on the external magnetic field present. Therefore, an element’s anisotropic shift is a vector quantity oriented in the same direction as the external magnetic field. Therefore, unlike isotropic shift values, anisotropic shift values are not averaged between specific values.

Tetra Methyl Silane (TMS) has a highly symmetric structure and has only a single peak in its NMR spectrum. Whether the NMR spectrum of a compound is taken experimentally or theoretically in a solvent, the resulting average isotropic shift values are calibrated by taking the single peak in the NMR spectrum of the TMS compound as reference. TMS is the most suitable reference material compared to some other substances in terms of its chemical and physical properties [49].

Since the theoretical NMR spectra of the compounds were obtained in water solvent medium, their isotropic average chemical protection values were calibrated by taking TMS as reference material for C and H elements, NH_3_ as reference material for N element and H_2_O as reference material for O element [27, 50].

**Figure 9:**
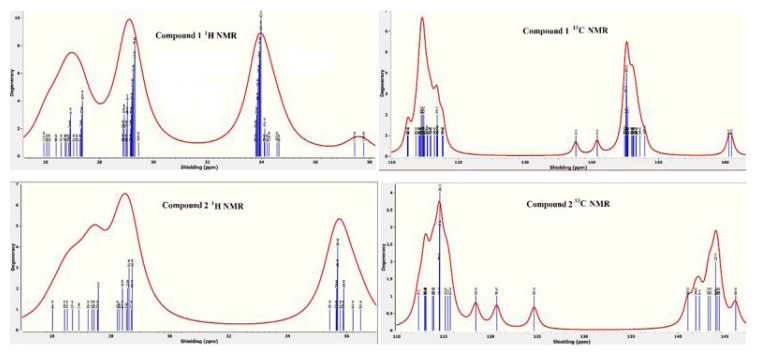
The ^1^H and ^13^C NMR spectra of compounds

Theoretical NMR calculations of the compounds were performed using the Gaussian 03 program, and for illustrative purposes, the ^1^H and ^13^C NMR graphs of the compounds are shown in Figure 9. The relevant NMR results are listed in Table S6.

The following conclusions can be obtained from examining the NMR spectra (Figure 9) of the compounds and Table S6.

- The elements that make up a compound can have either + or – chemical shielding values depending on their location, electrical interactions, and the type with amount of charge.
- The elements with the most positive or negative chemical shielding values in compounds are O with Ni in compound 1, and N with Ni in compound 2, respectively.
- The degeneracy numbers of H in compounds 1 and 2 vary between 1 and 10 with 1 and 4, respectively.
- The chemical shielding value of H atoms in compounds is generally ordered from greatest to least as follows: H_guest_ > H_amino_ > H_ring_.
- The degeneracy numbers of the C element in compounds 1 and 2 vary between 1 and 4 in both compounds.
- Compounds 1 and 2 are composed of a total of 86 and 62 individual or grouped atoms, respectively, of the same type or with different properties.
- The chemical shielding value of N atoms in compounds 1 and 2 is generally ranked from greatest to least as follows: N_amino_ > N_pyridine_ > N_cyano_. However, these values are even greater for the N atoms of the guest molecule than for the atoms of the ligand molecule (in compound 2).
- In compounds, the chemical shielding value of the C element in a molecule generally increases as the ring structure moves away from the neighboring N element. This can be explained by the fact that the N element in compounds exerts a greater pulling force on the protons than the C element.

### 4.7. Hirshfeld surface analyses of compounds

Long- and short-range interactions occurring between various atoms play important roles in the formation of a crystalline compound. The intensities of short-range interactions in a compound are significantly smaller than the intensities of long-range interactions. However, both groups of interactions are almost equally important for the formation of crystalline structures. Spackman and colleagues developed the CrystalExplorer program [51], which calculates short-range interactions in compounds using data from cif files.

The CrystalExplorer program calculates the different surface types of a molecular structure and various data related to these surfaces [51]. A 3D Hirshfeld surface of a compound can be visualized from the cif file by CrystalExplorer. Furthermore, a 2D “fingerprint” map of the compound in question is generated from this 3D Hirshfeld surface by the CrystalExplorer program. From a compound’s 2D fingerprint map, crucial information describing the interactions between neighboring atoms in its structure is obtained [51].

This program calculates various surface types of a molecular structure, the volumes and areas of their cavities, radii of curvature, electrical energies, and various properties [51]. However, CrystalExplorer cannot analyze the energy states of polymeric crystal structures.

**Figure 10:**
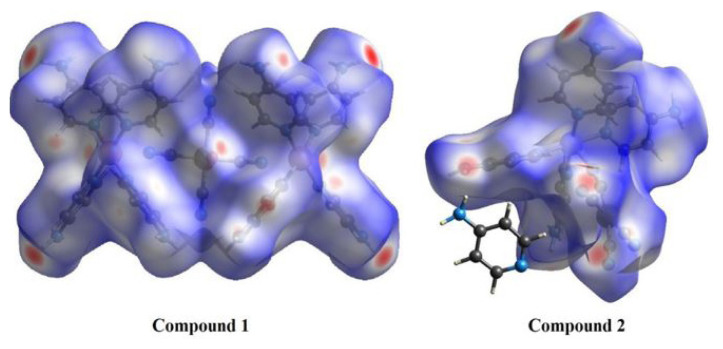
Views of the d_norm_ forms of the Hirshfeld surfaces of the compounds.

The red, blue, and white regions on a compound’s Hirshfeld surface represent hydrogen bonding interactions, long-range interactions, and van der Waals interactions, respectively [51]. The intensities of the respective colors are directly proportional to the strengths of the interactions shown. Hirshfeld surfaces of the compounds’ d_norm_ states, sample fingerprint plots, and various intermolecular contact values are presented in Figures 10, S6, and Table S7, respectively.

An analysis of Figure S6 and Table S7 reveals that the stabilization of the crystal lattice in both complexes is primarily driven by H···H, C···H, and H···N contacts, in descending order of significance. The topological similarity observed in the 2D fingerprint plots suggests a consistent packing motif; this is a direct consequence of both compounds possessing an identical stoichiometric ratio of 4AP ligands within their respective frameworks.

As evidenced by the data, the minority contributors to the lattice energy in complex 1 are the N···N, C···O, and Cu···N contacts. In contrast, for complex 2, the minimal interactions are represented by Ni···Ni, C···N, and N···Zn sites. The distinct nature of these forces is primarily governed by the identity of the central transition metal, leading to the observed variations between the two systems. Nevertheless, it is crucial to recognize that even these marginal interactions possess fundamental significance; they collectively cooperate to achieve the thermodynamic minimum, ensuring the overall stability of the crystalline architectures.

**Figure 11:**
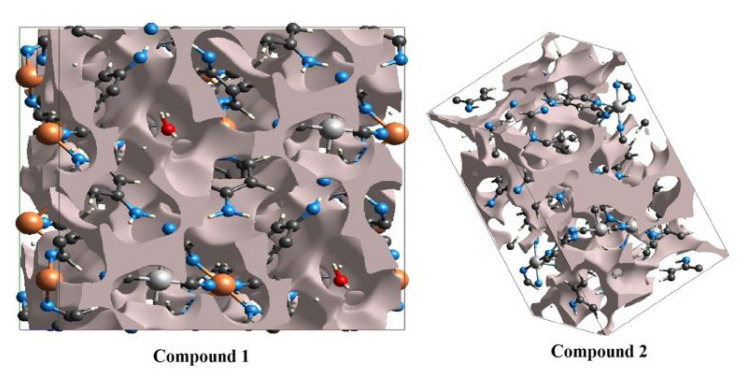
3D views of the crystal void of compounds.

To evaluate the porosity and free volume attributes of the crystalline frameworks, numerical simulations were performed utilizing the CrystalExplorer program. The crystal voids in the compounds are shown in Figure 11, respectively, in 3D. For easier comparison of the void properties of the compounds, the relevant data are presented in Table 7.

Examination of Table 7 shows that the void values of compound 1 are greater than those of compound 2.

The durability of a crystal lattice is defined by its capacity to resist mechanical perturbation. High porosity and excessive interstitial volumes are typically correlated with diminished packing efficiency, rendering the framework vulnerable to structural degradation even under minimal pressure. Therefore, maintaining sufficient mechanical stability is a critical prerequisite for frameworks designed for gas sequestration and guest molecule encapsulation.

Crystallographic analysis reveals that both compounds possess extended polymeric frameworks. Due to the inherent computational limitations of CrystalExplorer in evaluating non-discrete periodic systems, the determination of intermolecular interaction energies was not feasible for these specific architectures [51].

## 5. Conclusion

In this study, two new heteronuclear Hofmann-type-like clathrates, formulae [Cu(II)(4AP)_4_Ni(μ-CN)_2_(CN)_2_]·H_2_O (1) and [Zn(II)(4AP)_4_Ni(μ-CN)_2_(CN)_2_]·(4AP) (2) were successfully synthesized and characterized through a comprehensive suite of experimental and theoretical methods. The structural elucidation via SC-XRD and FT-IR confirmed that both compounds form 1D coordination polymer chains bridged by cyanonickelate units. Notably, while the host frameworks exhibit high structural similarity, the identity of the transition metal and the nature of the guest molecules (H_2_O vs. 4AP) lead to distinct physicochemical profiles. The comparative vibrational analysis highlighted the dual role of 4-aminopyridine in complex 2 as both a ligand and a guest, evidenced by the characteristic splitting of ν(NH_2_) stretching modes a feature absent in the single-role ligand environment of complex 1. Theoretically, DFT calculations (B3LYP/LanL2MB) provided deep insights into the electronic stability of the frameworks. The larger energy gap (ΔE) observed for the Zn-based clathrate (2) suggests higher kinetic stability and lower chemical reactivity compared to its Cu-based counterpart. Furthermore, the investigation into nonlinear optical (NLO) properties revealed that both clathrates possess remarkably high first-order static hyperpolarizability (β_0_) values approximately 49 and 158 times greater than that of the standard urea molecule for 1 and 2, respectively. These findings, supported by MEP and Hirshfeld surface analyses which identified H···H, C···H, and H···N contacts as the primary stabilizing intermolecular forces, position these materials as promising candidates for advanced optoelectronic applications. Collectively, this research demonstrates that the strategic selection of guest molecules and transition metals in Hofmann-type-like architectures offers a robust pathway for tuning the stability, porosity, and NLO responses of coordination polymers. It is thought that these compounds, due to some important structural and superior NLO properties, will be the subject of new studies in the future.

## Supplementary materials

Figure S1 The infinite 2D layer structure and $R_{2}^{1}(20)$ rings in compound 1.

Figure S2 Crystal structure of compound 2, showing the formation of C(12) chain.

Figure S3 Crystal structure of compound 2, showing the formation of $R_{2}^{2}(24)$ ring.

Figure S4 Crystal structure of compound 2, showing the formation of $R_{4}^{4}(36)$ ring.

Figure S5 The formation of a chain along [101] generated by C-H···π interactions in compound 2.

Figure S6 2D fingerprint plots of the three most effective interatomic contacts of the compounds.

**Table S1:** Elemental analysis values of Hofmann-type-like clathrates.

Compounds; M _r_ (g)	Elemental analysis, found(%)/(calculated)(%)					
	C	H	N	Ni	Cu	Zn
[Cu(C _5_H _6_N _2_) _4_Ni(CN) _4_]·H _2_O; Mr = 620.82	45.52 (46.44)	3.94 (4.22)	26.85 (27.08)	9.21 (9.46)	10.39 (10.24)	- (−)
[Zn(C _5_H _6_N _2_) _4_Ni(CN) _4_]·(C _5_H _6_N _2_); Mr = 698.75	49,09 (49.85)	4.07 (4.33)	27.82 (28.06)	8.05 (8.40)	- (−)	9.11 (9.36)

**Table S2:** Crystal data and structure refinement parameters of Hofmann-type-like clathrates.

Crystal data	1 *	2
Empirical formula	C _24_H _26_N _12_OCuNi	C _29_H _30_N _14_NiZn
Formula weight	620.82	698.75
Crystal system	Monoclinic	Monoclinic
Space group	*C2/c*	*P*2 _1_/ *n*
*a* (Å)	20.345 (4)	10.5067 (17)
*b* (Å)	17.357 (4)	23.200 (4)
*c* (Å)	19.452 (5)	13.306 (2)
*b* (°)	121.508 (13)	97.829 (5)
*V* (Å ^3^)	5857.0 (2)	3213.2 (9)
Z	8	4
*D* _c_ (g cm ^−3^)	1.408	1.444
θ range (°)	3.1–28.5	2.3–21.2
Measured refls.	69180	54075
Independent refls.	5701	6576
*R* _int_	0.069	0.104
S	1.34	1.05
R1/wR2	0.126/0.229	0.061/0.128
Dr _max_/Dr _min_ (eÅ ^−3^)	1.26/−1.85	0.47 / −0.65
CCDC	1565193	2487205

* This column refer to compound with CCDC number 1565193 from reference [15].

**Table S3:** Types of Mulliken electric charges of the atoms forming the compounds and their values according to the free electron charge (e)

Compound 1							
Atom	Charge	Atom	Charge	Atom	Charge	Atom	Charge
1 C	0.028629	31 H	0.122446	61 N	−0.520843	91 H	0.116881
2 C	0.086501	32 C	0.011479	62 H	0.271205	92 C	−0.142249
3 C	0.027702	33 H	0.167839	63 H	0.274870	93 H	0.101883
4 C	0.085144	34 C	−0.137741	64 C	−0.004161	94 C	0.142428
5 C	0.007418	35 H	0.125537	65 H	0.119638	95 C	−0.133676
6 H	0.150054	36 C	0.137501	66 C	−0.149709	96 H	0.119755
7 C	−0.139115	37 C	−0.147414	67 H	0.101863	97 C	0.015754
8 H	0.113692	38 H	0.101171	68 C	0.132998	98 H	0.150374
9 C	0.138323	39 C	−0.011476	69 C	−0.135230	99 N	−0.201944
10 C	−0.144281	40 H	0.115840	70 H	0.125478	100 N	−0.525700
11 H	0.104944	41 Cu	0.051764	71 C	0.012850	101 H	0.269910
12 C	−0.002542	42 Cu	0.042055	72 H	0.156621	102 H	0.268483
13 H	0.120288	43 N	−0.301225	73 C	0.004255	103 C	−0.004910
14 C	−0.005805	44 N	−0.245951	74 H	0.155252	104 H	0.118394
15 H	0.118221	45 N	−0.292606	75 C	−0.138774	105 C	−0.149898
16 C	−0.146455	46 N	−0.250002	76 H	0.114372	106 H	0.103479
17 H	0.101721	47 N	−0.209567	77 C	0.137269	107 C	0.140020
18 C	0.136043	48 N	−0.210546	78 C	−0.146818	108 C	−0.138181
19 C	−0.136633	49 N	−0.200287	79 H	0.104407	109 H	0.116286
20 H	0.123526	50 N	−0.206076	80 C	−0.003844	110 C	−0.003134
21 C	0.009181	51 Ni	−0.678850	81 H	0.119255	111 H	0.157787
22 H	0.161608	52 N	−0.522069	82 N	−0.209362	112 N	−0.206824
23 C	0.004732	53 H	0.278969	83 N	−0.209136	113 N	−0.519579
24 H	0.140822	54 H	0.276741	84 N	−0.525173	114 H	0.277826
25 C	−0.136367	55 N	−0.514794	85 H	0.272061	115 H	0.274734
26 H	0.112495	56 H	0.269926	86 H	0.275728	116 O	−0.404667
27 C	0.145934	57 H	0.273435	87 N	−0.514763	117 H	0.245597
28 C	−0.144107	58 N	−0.522366	88 H	0.275181	118 H	0.207850
29 H	0.105405	59 H	0.277338	89 H	0.273599		
30 C	−0.001936	60 H	0.271330	90 C	−0.003307		
**Compound 2**							
**Atom**	**Charge**	**Atom**	**Charge**	**Atom**	**Charge**	**Atom**	**Charge**
1 C	0.018311	20 H	0.123102	39 C	0.04	58 H	0.312303
2 H	0.165527	21 C	−0.1343	40 C	0.024227	59 H	0.306781
3 C	−0.1243	22 H	0.118769	41 N	−0.22383	60 N	−0.56155
4 H	0.137855	23 C	0.163225	42 N	−0.22044	61 H	0.309792
5 C	0.158332	24 C	−0.12052	43 N	−0.21929	62 H	0.305016
6 C	−0.13679	25 H	0.133766	44 N	−0.21529	63 C	−0.01074
7 H	0.120583	26 C	0.018355	45 N	−0.29837	64 H	0.150942
8 C	0.000709	27 H	0.162896	46 N	−0.28541	65 C	−0.15082
9 H	0.12403	28 C	0.002912	47 N	−0.25971	66 H	0.10346
10 C	−0.00573	29 H	0.126197	48 N	−0.29593	67 C	0.120029
11 H	0.118122	30 C	−0.13338	49 Ni	−0.6656	68 C	−0.15145
12 C	−0.1371	31 H	0.121068	50 Zn	0.411675	69 H	0.096262
13 H	0.116982	32 C	0.163026	51 N	−0.57593	70 C	−0.02392
14 C	0.161593	33 C	−0.12391	52 H	0.312977	71 H	0.111844
15 C	−0.12326	34 H	0.128624	53 H	0.309483	72 N	−0.21853
16 H	0.140827	35 C	0.014893	54 N	−0.56177	73 N	−0.58337
17 C	0.017836	36 H	0.152537	55 H	0.303541	74 H	0.278407
18 H	0.168462	37 C	0.129899	56 H	0.311416	75 H	0.28233
19 C	0.003549	38 C	0.0254	57 N	−0.56663		

**Table S4:** Some thermochemical data of compounds.

Thermochemical properties	Components	1	2
E (kcal/mol)	Electronic	0	0
	Translational	0.889	0.889
	Rotational	0.889	0.889
	Vibrational	648.586	417.171
	Total	650.364	418.948
Heat capacity at constant volume C _V_ (cal/mol-Kelvin)	Electronic	0	0
	Translational	2.981	2.981
	Rotational	2.981	2.981
	Vibrational	155.041	105.013
	Total	161.003	110.974
Entropy S (cal/mol-Kelvi)	Electronic	0	0
	Translational	46.75	45.502
	Rotational	41.475	39.075
	Vibrational	172.905	121.472
	Total	261.13	206.048
Zero-point vibrational energy E _v0_	(Joules/mol)	2605995	1671721
	(kcal/mol)	622.848	399.551
Rotational constants (GHz)	A	0.04397	0.07228
	B	0.01662	0.04355
	C	0.0154	0.04005

**Table S5:** List of some properties of theoretical excitation states that provide the greatest contribution to the UV-visible excitation states of compounds.

Compounds	Excitation states	Excitation energy (eV)	Wavelength (nm)	Oscillation strengths (f)	Assignments From → to	Coefficients (C _i_)	Contribution (%)
1	1	14.099	0879.39	0.0004	259→260	0.70205	0.9857484
	2	14.495	0855.38	0.0002	258→260	0.70168	0.9847096
	3	20.159	0615.04	0.0020	258→261	0.66845	0.8936508
	4	20.403	0607.68	0.0004	259→262	0.61892	0.7661239
	5	21.912	0565.82	0.0047	259→263	0.68195	0.9301116
	6	21.993	0563.75	0.0066	258→264	0.57487	0.6609510
	7	22.322	0555.45	0.0011	259→265	0.65308	0.8530269
	8	22.505	0550.92	0.0004	258→267	0.55027	0.6055941
	9	22.585	0548.96	0.0006	259→266	0.52267	0.5463678
	10	22.739	0545.24	0.0007	259→271	0.51906	0.5388465
	11	22.926	0540.80	0.0002	259→268	0.65864	0.8676132
	12	23.529	0526.95	0.0001	258→269	0.62650	0.7850045
	13	23.631	0524.66	0.0021	258→270	0.51609	0.5326977
	14	23.798	0520.99	0.0005	259→272	0.42205	0.3562524
	15	24.406	0508.00	0.0004	258→273	0.54166	0.5867911
	16	24.478	0506.52	0.0011	259→277	0.62815	0.7891448
	17	25.040	0495.15	0.0002	258→275	0.56966	0.6490250
	18	25.367	0488.77	0.0001	258→276	0.66167	0.8756143
	19	25.585	0484.60	0.0002	259→274	0.36601	0.2679266
	20	25.880	0479.06	0.0013	258→278	0.33186	0.2202621
2	1	44.536	0278.39	0.0025	161→176	0.62656	0.7851548
	2	45.502	0272.48	0.0007	160→176	0.62371	0.7780283
	3	45.656	0271.56	0.0010	166→167	0.70249	0.9869844
	4	45.948	0269.84	0.0185	165→167	0.69607	0.9690268
	5	46.101	0268.94	0.0005	158→176	0.57268	0.6559247
	6	47.513	0260.95	0.0003	155→176	−0.55820	0.6231744
	7	49.182	0252.09	0.1082	161→167	0.67051	0.8991673
	8	49.616	0249.89	0.0003	166→168	0.67530	0.9120601
	9	49.674	0249.60	0.0255	160→167	0.66153	0.8752438
	10	50.354	0246.22	0.0401	158→167	0.63858	0.8155688
	11	51.155	0242.37	0.0008	165→168	0.69340	0.9616071
	12	51.632	0240.13	0.0034	157→167	0.67696	0.9165496
	13	52.596	0235.73	0.0001	166→170	0.53012	0.5620544
	14	52.824	0234.71	0.0009	164→167	0.68445	0.9369436
	15	53.109	0233.45	0.0065	156→167	0.47616	0.4534566
	17	53.671	0231.01	0.0015	163→167	0.50314	0.5062997
	18	53.699	0230.89	0.0001	166→172	0.46173	0.4263891
	19	53.788	0230.51	0.0007	165→178	−0.68510	0.9387240

**Table S6:** The NMR theoretical Isotropic and Anisotropic chemical and magnetic shielding values calculated for compounds in water solvent at room temperature.

Atoms	Elements	Degeneracy	Chemical and Magnetic shielding values (ppm)		
			Average	Shielding value corrected to ref.	Anisotropic
**Compound 1**					
51	Ni	1	−2570,63	-	1819,9454
42	Cu	1	−393,162	-	338,8785
45	N	1	16,319	242,081	411,1397
43	N	1	21,626	236,774	406,268
111	H	1	25,934	5,9481	10,8682
33	H	1	26,042	5,8401	8,7186
22	H	1	26,131	5,7511	9,7465
98	H	1	26,385	5,4971	11,306
72	H	1	26,57	5,3121	11,0517
40	H	1	26,715	5,1671	10,1251
91	H	1	26,769	5,1131	11,6159
74;24;6	H	3	26,8893	4,99276	12,2841;13,0537;12,1562
31	H	1	27,041	4,8411	13,9912
65	H	1	27,153	4,7291	11,4724
81;13;15;104	H	4	27,3162	4,56585	12,4083;13,1624;11,3178;11,3636
70;96;109	H	3	28,8773	3,00476	6,5532;5,8023;8,1099
35;20;38;8	H	4	29,0025	2,8796	7,2541;7,7925;6,0688;7,6358
26;11;76;93; 67;29;17;79	H	8	29,2115	2,6706	8,3955;7,2412;8,3638;6,8791; 6,4049;6,9734;6,4642;6,783
106	H	1	29,438	2,4441	6,2559
57;89;53; 85;86;88;54; 56;114;62	H	10	33,8784	−1,9963	18,8464;19,5689;18,1808; 18,5414;17,5408;18,3975;18,7938; 20,0668;18,422;19,6977
59;101	H	2	34,1035	−2,2214	20,7831;19,8401
63	H	1	34,202	−2,3199	18,8785
115	H	1	34,275	−2,3929	19,8711
102	H	1	34,568	−2,6859	19,4572
60	H	1	34,638	−2,7559	21,3546
117	H	1	37,447	−5,5649	25,5113
118	H	1	37,782	−5,8999	17,6226
44	N	1	80,347	178,053	326,67
46	N	1	84,954	173,446	327,2855
97	C	1	112,341	70,1246	135,1435
30	C	1	112,455	70,0106	127,4097
80	C	1	113,598	68,8676	128,9941
90	C	1	114,015	68,4506	130,8876
21	C	1	114,197	68,2686	132,8819
12	C	1	114,266	68,1996	127,2527
23;39	C	2	114,412	68,0536	132,0844;126,1335
64;5	C	2	114,5515	67,9141	129,5861;133,114
110	C	1	114,672	67,7936	133,0528
68;71	C	2	114,7675	67,6981	141,7493;133,295
9	C	1	114,926	67,5396	140,0694
103	C	1	115,276	67,1896	130,2332
32	C	1	115,386	67,0796	134,2913
14	C	1	115,759	66,7066	126,214
73	C	1	115,839	66,6266	130,9936
94	C	1	116,356	66,1096	140,8639
36;27	C	2	116,7555	65,7101	140,2492;137,9834
107	C	1	116,869	65,5966	140,2574
18	C	1	117,565	64,9006	137,5666
77	C	1	117,645	64,8206	138,1787
99	N	1	118,757	139,643	343,1656
49	N	1	120,819	137,581	340,3213
82	N	1	125,082	133,318	342,4492
47	N	1	125,297	133,103	341,5679
83	N	1	128,121	130,279	337,712
112	N	1	129,133	129,267	336,6939
48	N	1	130,72	127,68	334,4029
50	N	1	131,959	126,441	331,2162
2	C	1	137,582	44,8836	226,7719
4	C	1	140,779	41,6866	224,1819
25	C	1	144,9	37,5656	133,4298
108;7;37;92	C	4	145,1097	37,35585	131,4021;133,4585;132,0498;134,9216
10	C	1	145,304	37,1616	135,309
69	C	1	145,354	37,1116	135,1787
66	C	1	145,405	37,0606	134,013
19	C	1	145,924	36,5416	129,7743
75	C	1	146,083	36,3826	130,0338
16	C	1	146,135	36,3306	129,8425
78	C	1	146,295	36,1706	133,8263
28	C	1	146,592	35,8736	129,7649
95	C	1	146,673	35,7926	130,338
34	C	1	147,196	35,2696	128,8193
105	C	1	147,917	34,5486	130,8042
3	C	1	160,543	21,9226	189,1472
1	C	1	160,934	21,5316	187,6511
61	N	1	279,052	−20,652	65,6263
113;52;84;55	N	4	279,294	−20,894	66,4675;78,5249;77,2535;67,1226
87	N	1	279,436	−21,036	67,7828
58	N	1	282,213	−23,813	35,7051
100	N	1	282,325	−23,925	34,5127
116	O	1	445,087	−125,087	70,5976
**Compound 2**					
49	Ni	1	−2669,125	-	1875.6258
47	N	1	13,623	244,777	405,3739
46	N	1	19,805	238,595	414,3627
48	N	1	19,93	238,47	400,2969
64	H	1	26,016	5,8661	6,1911
18	H	1	26,413	5,4691	9,116
71	H	1	26,507	5,3751	6,673
27	H	1	26,682	5,2001	11,1368
2	H	1	26,906	4,9761	11,3708
36	H	1	27,215	4,6671	11,1666
11	H	1	27,346	4,5361	11,231
29	H	1	27,421	4,4611	12,3151
20;9	H	2	27,551	4,3311	11,8307;11,3416
25	H	1	28,221	3,6611	6,9298
4	H	1	28,282	3,6001	7,8743
34;16	H	2	28,382	3,5001	7,5332;7,2432
7;13;31	H	3	28,561	3,3211	6,2311;6,1571;6,5526
22;69;66	H	3	28,704	3,1781	7,0126;5,7472;5,3506
75	H	1	35,426	−3,5439	19,3601
62;74;55;56	H	4	35,6682	−3,78615	20,4274;16,3248;19,8506;18,8581
61	H	1	35,784	−3,9019	19,3828
59;58	H	2	35,889	−4,0069	18,9074;20,1235
52	H	1	36,21	−4,3279	19,0623
53	H	1	36,473	−4,5909	19,1932
72	N	1	54,745	203,655	472,1132
5	C	1	112,288	70,1776	145,8013
28	C	1	112,925	69,5406	132,5626
32	C	1	113,02	69,4456	145,2927
14	C	1	113,073	69,3926	145,4173
19	C	1	113,764	68,7016	131,3566
23	C	1	113,888	68,5776	144,9099
10;35;8;26	C	4	114,5052	67,96035	131,2862;134,2414;132,4747;134,7845
17	C	1	115,118	67,3476	135,3323
1	C	1	115,363	67,1026	133,9193
67	C	1	115,637	66,8286	143,1503
63	C	1	118,353	64,1126	140,2513
70	C	1	120,565	61,9006	140,1911
37	C	1	124,53	57,9356	237,532
45	N	1	132,385	126,015	257,2744
33	C	1	140,778	41,6876	133,4142
24	C	1	141,6	40,8656	133,8271
3	C	1	141,999	40,4666	133,2509
15	C	1	142,946	39,5196	129,7332
12	C	1	143,161	39,3046	131,4048
6;21	C	2	143,71	38,7556	132,072;131,329
30	C	1	143,857	38,6086	131,479
68	C	1	144,082	38,3836	136,8342
65	C	1	145,838	36,6276	136,1462
44	N	1	146,99	111,41	287,2852
42	N	1	148,404	109,996	288,4221
41	N	1	149,036	109,364	283,7042
43	N	1	149,153	109,247	283,4804
39	C	1	162,31	20,1556	187,7258
40;38	C	2	163,013	19,4526	183,8624;184,9612
50	Zn	1	214,593	-	7,299
54	N	1	280,417	−22,017	69,4548
60	N	1	281,03	−22,63	62,0685
57	N	1	281,453	−23,053	67,8099
51	N	1	281,707	−23,307	69,1961
73	N	1	287,261	−28,861	83,8827

**Table S7:** Percentage contributions of various intermolecular contacts of compounds.

Intermolecular contacts	Compounds		Intermolecular contacts	Compounds	
	1	2		1	2
C···C	2.4	2.1	H···N/N···H	24.0	26.3
C···H/H···C	28.3	28.5	H···Ni/Ni···H	0.9	1.7
C···N/N···C	0.6	0.8	H···O/O···H	1.1	-
C···O/O···C	0.3	-	N···N	0.06	-
Cu···N/N···Cu	0.5	-	N···Ni/Ni···N	-	0.4
H···H	41.8	38.2	N···Zn/Zn···N	-	1.4

## Figures and Tables

**Table 1:** Selected bond distances and angles for compounds 1 and 2 (Å, °).

Compound 1				Compound 2			
Cu1-N5	2.026(7)	Cu1-N7	2.040(7)	Zn1-N1	2.093(4)	Zn1-N3	2.145(4)
Cu2-N9	2.029(7)	Cu2-N11	2.041(8)	Zn1-N5	2.114(4)	Zn1-N7	2.128(4)
Cu1-N2	2.692(4)	Cu1-N4	2.695(4)	Zn1-N9	2.113(4)	Zn1-N10^ii^	2.122(4)
Ni1-C1	1.855(10)	Ni1-C2	1.863(8)	Ni1-C21	1.867(5)	Ni1-C22	1.875(5)
Ni1-C3	1.848(11)	Ni1-C4	1.867(8)	Ni1-C23	1.869(6)	Ni1-C24	1.854(6)
N5-Cu1-N7	178.0(3)	N9-Cu2-N11	173.7(3)	N1-Zn1-N9	90.18(15)	N1-Zn1-N5	177.46(16)
				N9-Zn1-N5	91.58(15)	N1-Zn1-N7	89.84(15)
				N9-Zn1-N7	89.35(15)	N5-Zn1-N7	92.01(15)
				N1-Zn1-N3	88.97(15)	N9-Zn1-N3	89.03(15)
(i) x, y, −z+1/2; (iii) −x+1, y, −z+1/2				(ii) x−1/2, −y+1/2, z−1/2			

**Table 2:** Hydrogen bonds and C-H···π interaction parameters for compound 2 (Å, °).

D-H···A	D-H	H···A	D···A	D-H···A
N4—H4B···N11iii	0.81 (2)	2.41 (2)	3.216 (7)	172
N6—H6A···N12^iv^	0.81 (2)	2.46 (4)	3.188 (8)	150
N8—H8B···N13^v^	0.82 (2)	2.22 (2)	3.030 (7)	168
N14—H14A···N12^iv^	0.82 (2)	2.28 (3)	3.060 (8)	159
C4—H4···Cg3^vi^	0.93	2.66	3.5438 (6)	160

Symmetry codes:

iii −x+3/2, y−1/2, −z+3/2;

iv −x+1, −y+1, −z+1;

v x, y, z−1;

vi 1/2+x, 1/2-y, −1/2+z; Cg3=N5/C11-C15.

**Table 3:** Some vibration wavenumbers (cm^−1^) of 4AP in free state and in compounds 1 and 2.

Assignment a	Free 4AP	1*	Δ	2	Δ
ν_as_(NH_2_)	3430 vs	3479 m	**49**	3469 s 3424 m	**39** −6
ν_s_(NH_2_)	3303 w	3342 s	**39**	3367 s 3215 m	**64** **−88**
ν(C-H)	3079 w	3073 vw	−6	3063 w	−16
δ(NH_2_)	1649 m	1641 s, br	−8	1663 s 1634 s	14 −15
ν(C-C), γ(C-C-C)	1585 s	1609 m	24	1613 s	28
ν(C=C)	1526 m	1562 m	**36**	1564 s	**38**
ν(C=N)	1494 m	1520 s	26	1513 s	19
ν(C-C)	1429 w	1455 m	26	1445 m	16
ν(C-NH_2_), ν(C-C)	1329 w	1346 m	17	1383 m 1334 m	**54** 5
ν(C-N)	1267 m	1283 m	16	1274m	7
γ(C-H)	1204 m	1213 s	9	1213 s	9
γ(C-H), γ(C-C-C)	1055 w	1059 m	4	1060 m	5
γ(C-C-C), ring breathing	976 s	1022 s	**46**	1014 s	**38**
ω(NH_2_), τ(NH_2_)	838 sh	853 m	15	846 s	8
β(C-H)	813 vs	828 s	15	824 s	11
γ(C-C-C), ring deformation	664 s	669 vw	5	706 vw	**42**
γ(C-N-C), ω(ring), ρ(NH_2_)	524 s	531 s	7	532 s	8

ν: stretching; δ: bending; γ: in plane bending; β: out of plane bending; ω: wagging; τ: torsion; ρ: rocking; s: strong; m: medium; w: weak; vw: very weak; sh: shoulder.

a taken from [15].

* This column refer to compound with CCDC number 1565193 from reference [15].

**Table 4:** The IR-active vibration wavenumbers (cm^−1^) of the Ni(CN)_4_ ion in K_2_[Ni(CN)_4_]·H_2_O and compounds 1 and 2.

Assignment^a^	K_2_[Ni(CN)_4_]·H_2_O	1*	Δ	2	Δ
ν_8_(C≡N), E_u_	2122 vs	2170 vs (bridge) 2136 s (terminal)	48 14	2155 vs (bridge) 2124 vs (terminal)	33 2
ν_9_(Ni–CN), E_u_	544 w	565 w	21	560 sh	16
π(Ni–CN), A_2u_	442 w	448 w	6	-	-
δ(Ni–CN), E_u_	420 s	417 s	−3	421 s	1

ν: stretching, δ: in plane bending, π: out-of-plane bending, vs: very strong, s: strong, m: medium, w: weak, sh: shoulder.

* This column refer to compound with CCDC number 1565193 from reference [15].

a Taken from Ref. [25].

**Table 5:** The HOMO, LUMO and chemical efficiency values in (a.u.) and (eV) units of compounds.

Chemical efficiency values	1		2	
E_HOMO_ (−I)	−0.02257	−0.615	−0.09789	−2.664
E_LUMO_ (−A)	0.07962	2.166	−0.01129	−0.307
ΔE = E_LUMO_ – E_HOMO_	0.1022	2.781	0.0866	2.357
χ	0.0285	0.776	−0.0546	−1.486
μ	−0.0285	−0.776	0.0546	1.486
η	−0.0511	−1.391	−0.0433	−1.178
S (a.u.)^−1^, (eV)^−1^	−9.784	−0.359	−11.54	−0.424
ω	−0.0079	−0.216	−0.0344	−0.937

**Table 6:** The electric dipole moment, anisotropies of polarizability, the mean polarizability, the first- and second-order static hyperpolarizabilities of compounds.

Parameters	Compounds	
	1	2
μ (D)	12.6227	40.9904
Δα (esu)	1.622713×10^−23^	3.006419×10^−23^
α_0_ (esu)	−3.836820×10^−23^	−3.366212×10^−23^
β_0_ (esu)	3.748875×10^−30^	12.09084×10^−30^
γ (esu)	−11.065621×10^−36^	−5.536297×10^−36^

**Table 7:** General characteristics of the voids in the compounds.

Compounds	Volume (Å^3^)	RVVTV (%)	Void area (Å^2^)	Globularity	Asphericity
1	973.01	16.61	2762.04	0.172	0.103
2	451.73	14.06	1300.48	0.219	0.210

RVVTV (%). Ratio of void volume to total volume (%).
