# Supplementary figures and images for: Synthesis of two Hofmann-type-like clathrates, characterizations, and investigation of their structures, spectroscopic and theoretical properties
Source: Turk J Chem. 2026 Apr 9;50(3):341–58. doi: 10.55730/1300-0527.3802 (PMC13384572; doi:10.55730/1300-0527.3802)

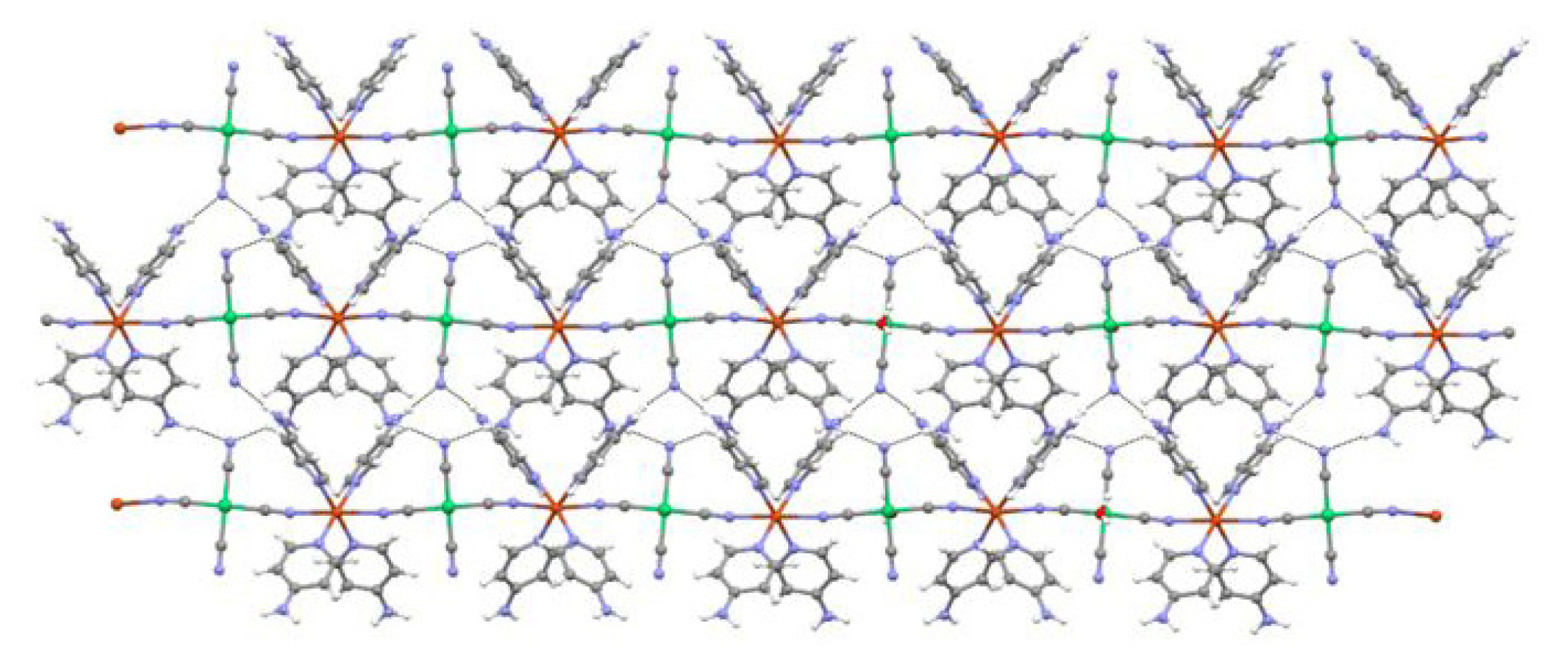

Supplement: Figure S1 — The infinite 2D layer structure and R21(20) rings in compound 1. [file tjc-50-03-341s1.tif]

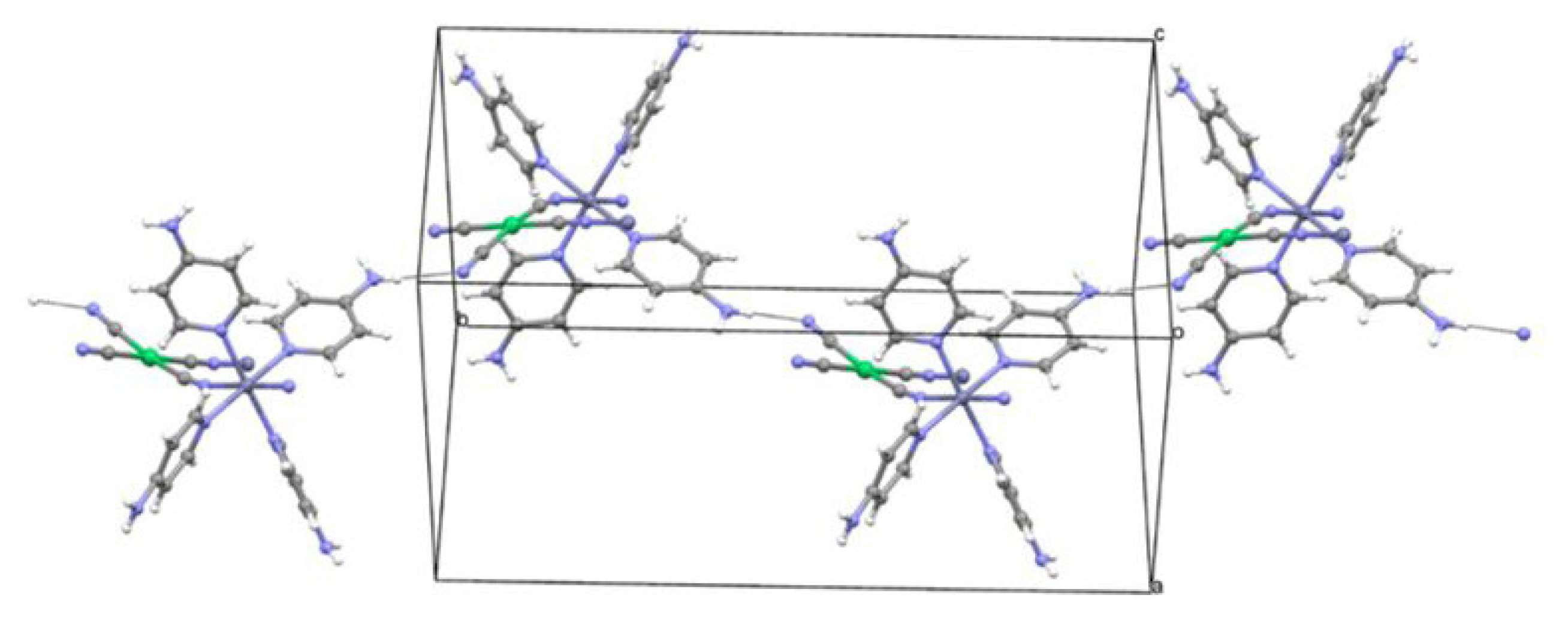

Supplement: Figure S2 — Crystal structure of compound 2, showing the formation of C(12) chain. [file tjc-50-03-341s2.tif]

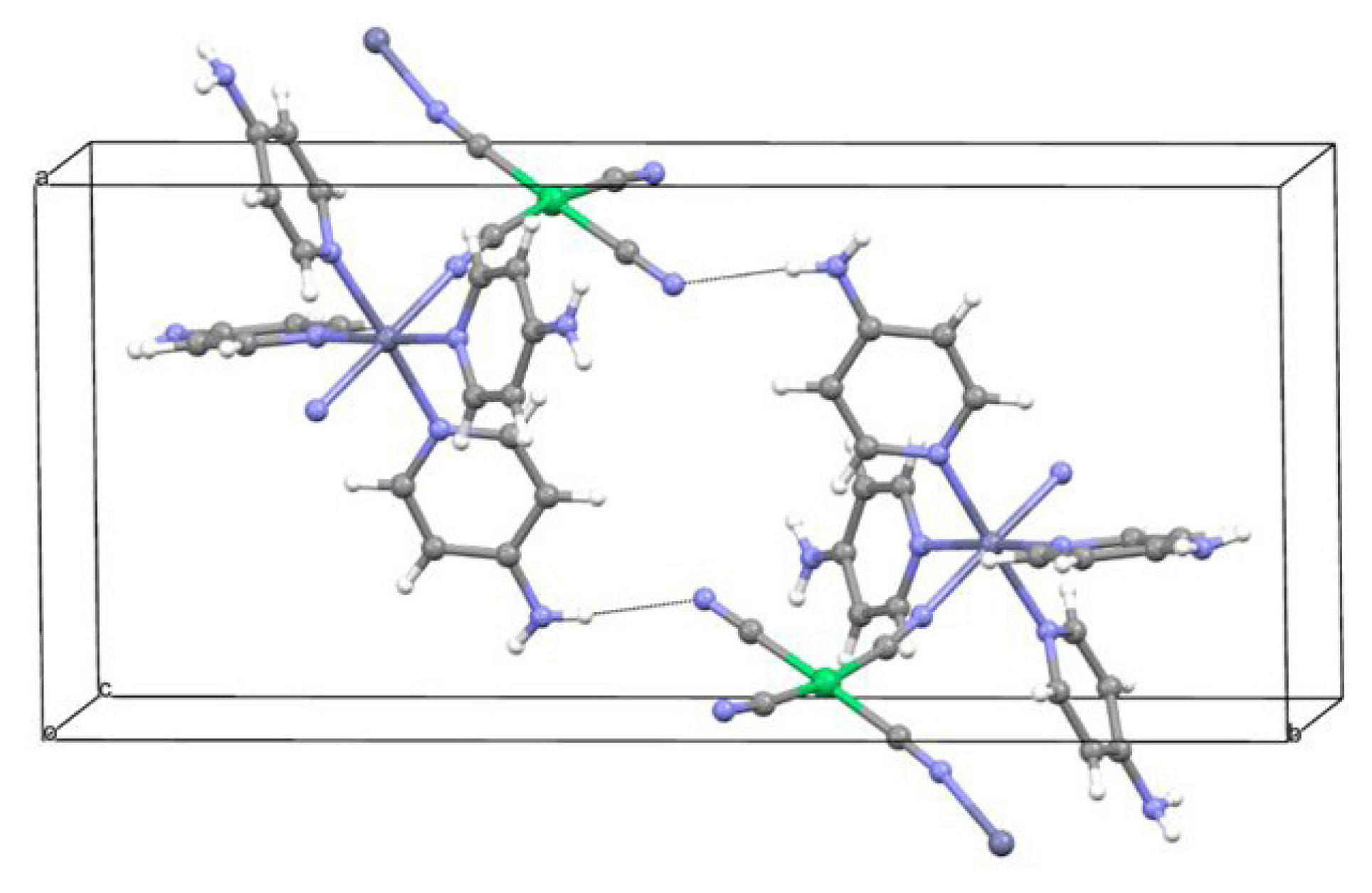

Supplement: Figure S3 — Crystal structure of compound 2, showing the formation of R22(24) ring. [file tjc-50-03-341s3.tif]

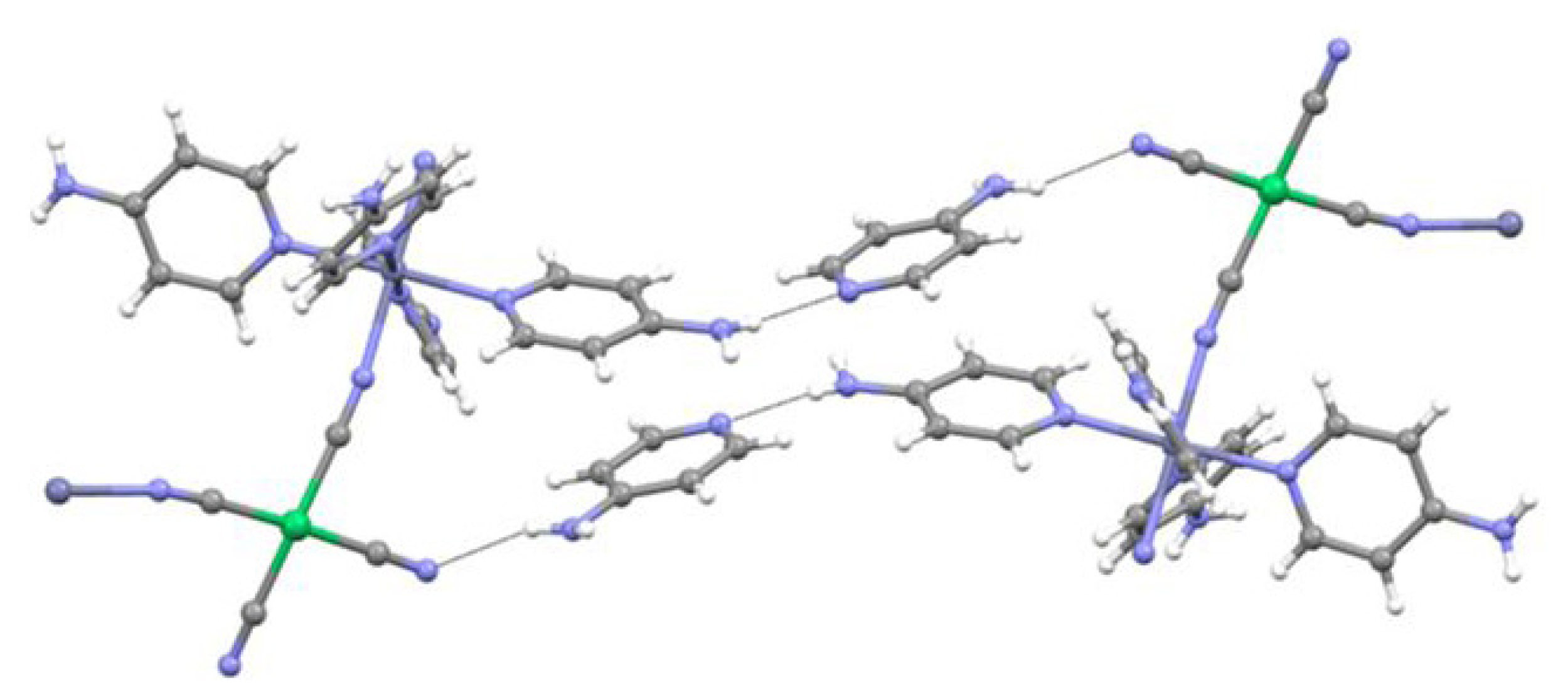

Supplement: Figure S4 — Crystal structure of compound 2, showing the formation of R44(36) ring. [file tjc-50-03-341s4.tif]

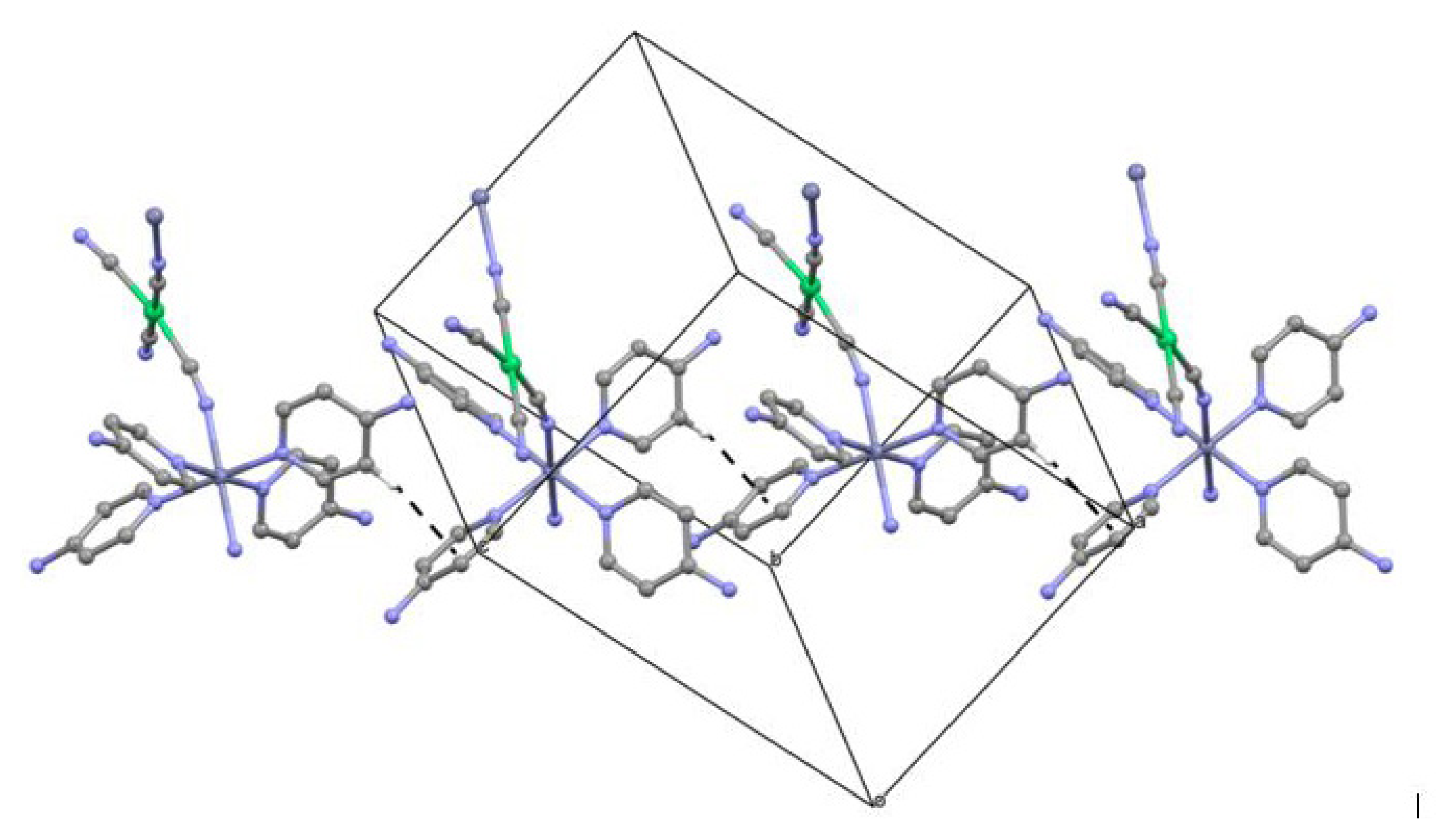

Supplement: Figure S5 — The formation of a chain along [101] generated by C-H···π interactions in compound 2. [file tjc-50-03-341s5.tif]

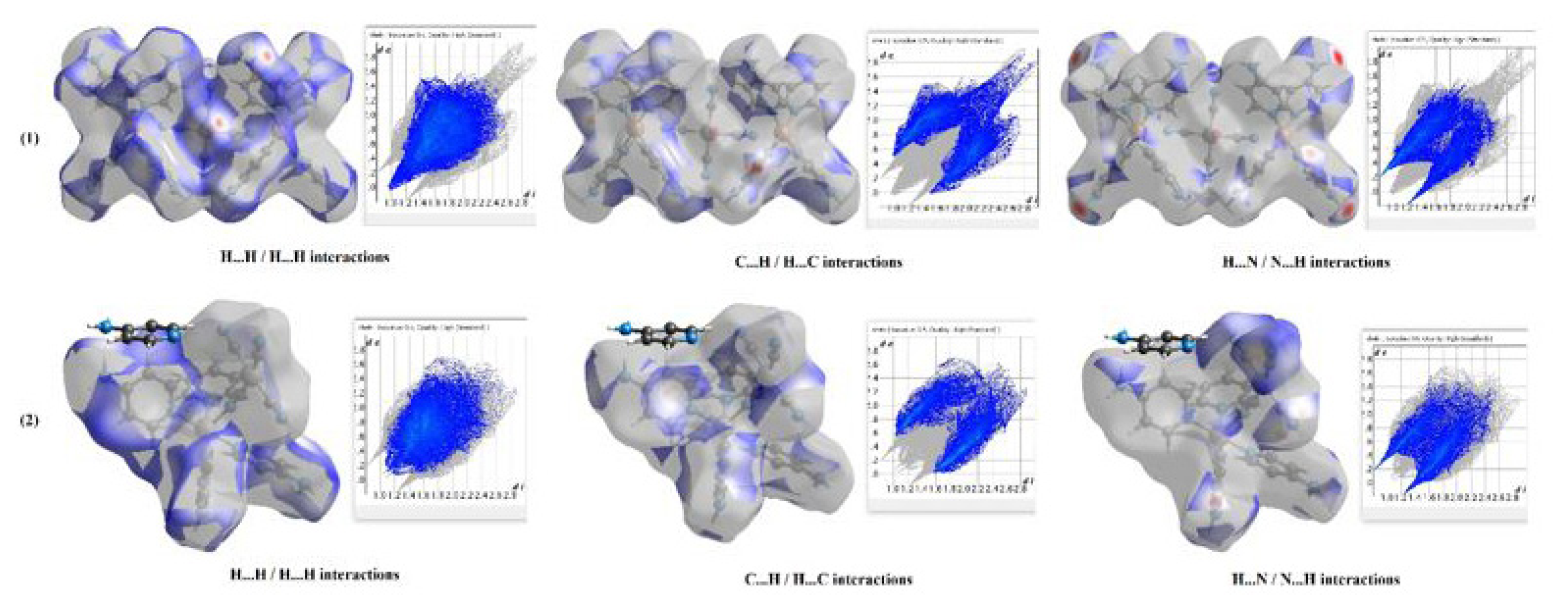

Supplement: Figure S6 — 2D fingerprint plots of the three most effective interatomic contacts of the compounds. [file tjc-50-03-341s6.tif]
